# Paravertebral Block Combined with Sedation for a Myasthenic Patient Undergoing Breast Augmentation

**DOI:** 10.1155/2015/593282

**Published:** 2015-08-17

**Authors:** Betul Kozanhan, Betul Basaran, Leyla Kutlucan, Sadık Ozmen

**Affiliations:** Department of Anesthesiology and Reanimation, Konya Training and Research Hospital, Meram Yeniyol Street No. 97, Meram, 42090 Konya, Turkey

## Abstract

Paravertebral block is a unilateral analgesic technique that can provide adequate surgical anesthesia and great advantages in many types of surgery with a low side-effect profile. In this case we present combination of bilateral thoracic paravertebral block under ultrasound guidance with sedation which provides complete anesthesia and postoperative analgesia in a myasthenic patient undergoing cosmetic breast surgery. In myasthenic patients paravertebral blocks may be a better option for breast surgery with avoiding the need for muscle relaxants and opioids and risk of respiratory failure in postoperative period.

## 1. Introduction

Thoracic paravertebral block (TPVB) is a regional anesthesia technique that involves injection of a local anesthetic alongside the thoracic vertebral body close to where the spinal nerves emerge from the intervertebral foramen [1]. This produces unilateral, segmental, somatic, and sympathetic nerve blockade in multiple adjacent thoracic dermatomes [1]. Combined with sedation, TPVB provides effective surgical anesthesia for patients undergoing oncological breast procedures and breast augmentation [2]. We report here the use of ultrasound-guided bilateral TPVB with sedation as a primary anesthetic technique, in a patient with myasthenia gravis (MG) undergoing bilateral cosmetic breast augmentation.

## 2. Case Presentation

A 28-year-old female (60 kg; 165 cm; ASA II) presented for bilateral cosmetic breast augmentation. Ten years prior to this presentation she had been diagnosed with MG with respiratory dysfunction and mild generalized weakness and classified as Osserman stage III. At the time of diagnosis she underwent thymectomy. After the operation tracheal intubation was performed and remained for 7 postoperative days due to myasthenic crisis. During preoperative evaluation her neurological examination was normal and there were no symptoms of MG. She was not receiving medication. Preoperative pulmonary function tests revealed her forced vital capacity (FVC) 3.51 L, with 1 s forced expiratory volume (FEV1) of 2.95 L. The patient's baseline vital signs were a heart rate of 76 bpm and blood pressure of 130/70 mmHg. After discussions with the patient about options for anesthesia a decision was made to proceed with TPVB.

In order to provide better consistency in the spread of the local anesthetic and produce a more reliable sensory block, we planned to perform multiple level injections TPVB [3]. On the day of surgery, standard ASA monitors were applied, intravenous access was established, and crystalloid infusion was started. While the patient was in the sitting position, Th2–Th5 spinous processes were identified and marked by palpating and counting down from vertebra prominens (C7). Under aseptic conditions, local anesthesia with 2% lidocaine was performed to skin and subcutaneous tissues. Linear array probe (Esaote MyLab 5, Genova, Italy) was placed longitudinally in sagittal plane at a point 2-3 cm lateral to the midline. Both transverse processes were visualized as two hyperechoic lines and the parietal pleura was visualized as a bright structure running deep to the adjacent transverse processes. If the pleura was not clearly delineated probe was slightly tilted laterally. The distance from the skin to the paravertebral space was measured (38, 41, and 43 mm at Th3, Th4, and Th5, resp.) and a 22-gauge, echogenic peripheric block needle (Pajunk, Germany) was inserted using in-plane technique in caudad to cephalad direction. The tip of needle was advanced until the superior costotransverse ligament punctured. The proper placement of the needle in the paravertebral space was confirmed with hydrolocation after negative aspiration of blood or air local anesthetic was slowly injected. Spread of local anesthetic was visualized with displacement of the pleura anteriorly. The same procedure was repeated at both bilateral Th2–Th5 levels and total 30 mL of 0.5% levobupivacaine was divided equally in paravertebral spaces. During the block procedure verbal contact was maintained with the patient and there were no signs of local anesthetic toxicity. Also no difficulty of breath or desaturation occurred. Loss of sensation to cold and pinprick of chest wall from Th2 to Th6 dermatomes was verified 20 min following block placement. Intraoperative sedation (Ramsay score of 2-3) was provided with continuous infusion of propofol 30–50 mcg/kg/min (total given was 266 mg). The patient remained hemodynamically stable throughout the 115 min of surgery. At the end of the procedure she was comfortable and pain management consisted of oral acetaminophen 500 mg every 6 h as the postoperative routine. She remained pain-free overnight and did not require any opioids for analgesia. She received tenoxicam 20 mg intravenous for a pain score of 4 (visual analog scale (0 = no pain and 10 = the worst pain)) at the postoperative 15 h. Her lung function capacity was assessed after 24 h and FVC was 3.12 L. She was discharged home on the second postoperative day without any complications.

## 3. Discussion

Myasthenia gravis (MG) is an autoimmune neuromuscular disease characterized by a decrease in acetylcholine receptors secondary to their destruction or inactivation by circulating IgG antibodies causing weakness and fatigue in ocular, bulbar, limb, and respiratory muscles due to repetitive use [4]. Anesthesia in patients with MG requires special attention, because of an abnormal response to muscle relaxants, increased sensitivity to sedatives, and a restricted respiratory capacity [5, 6]. These patients are more sensitive to the effects of nondepolarizing neuromuscular blockers and resistant to the effects of succinylcholine [4, 5]; therefore some clinicians avoid muscle relaxants and prefer deep inhalational anesthesia for facilitating tracheal intubation. However, inhalational anesthetics would be a factor for depression of neuromuscular function in myasthenic patients [5]. Asymptomatic myasthenic patients should be assumed to be sensitive to the effect of neuromuscular blocking agents and volatile anesthetics [6]. In addition, effectively blocking pain pathways is important to reduce the acute surgical stress response which can worsen disease symptoms and may trigger myasthenic crisis [4, 5]. Postoperative respiratory compromise may be associated with pain. In this way opioid usage could be a factor for central respiratory depression. So relatively opioid free anesthetic modalities are accepted and convenient for this type of patients [4, 5].

TPVB can be used with excellent effect for analgesia but it is also suitable as a sole anesthetic technique. Studies have shown that paravertebral block has been associated with an improved postoperative pain scores with reduced requirements for opioid analgesics, decreased incidence of nausea and vomiting, quicker discharge times, and greater patient satisfaction when compared to general anesthesia [2, 7]. Bilateral TPVB may cause blockade of intercostal muscles. This leads to concern about respiratory depression in myasthenic patients. However, when compared to thoracic epidural analgesia, TPVB provides comparable analgesia with epidural blockade and is associated with a significantly better postoperative pulmonary function, earlier mobilization, less urinary retention, and hypotension [8, 9].

Paravertebral block is generally associated with low and acceptable side effects and complications. Hypotension, theoretic high blood concentrations of local anesthetic, and epidural or spinal spread of local anesthetic and pneumothorax are the possible controversial issues related to the bilateral PVB [10]. Ultrasound has been used to improve efficacy and reduce complications via real-time visualization of the intended anatomic space, surrounding structures, and the approaching needle. Though bilateral PVB caused an eightfold increase in the rate of the pneumothorax [11], the study using in-plane ultrasound technique did not indicate any pleural puncture from paravertebral block [12]. We performed TPVB under ultrasound guidance and measured the distance from the skin to paravertebral space to potentially minimize the risk of pneumothorax. We used multiple injections slowly with low doses of local anesthetic at each level to prevent the spread of anesthetic from paravertebral to epidural space. Beside these we also used low dose amide local anesthetic levobupivacaine because its metabolism is independent of pseudocholinesterase function with long-term continuing analgesia into the postoperative period. Our patient's postoperative respiratory function has limited deterioration possibly due to used opioid sparing anesthetic technique.

We demonstrate that administering bilateral TPVB is a safe and useful procedure for performing cosmetic breast augmentation for patients when general anesthesia is not desirable. The procedure is advantageous in that the use of muscle relaxants, volatile anesthetic agents can be avoided. The risk of perioperative respiratory depression caused by high dose opioid usage can be minimized. Performing the block with ultrasound guidance by an anesthesiologist who is experienced in this technique, the chance of pneumothorax will be extremely low.
